# The clinical impact of endoscopic ultrasound-guided fine-needle aspiration on the patients with low-risk pancreatic cystic lesions

**DOI:** 10.3389/fonc.2022.961293

**Published:** 2022-08-05

**Authors:** Shubo Pan, Jie Liu, Jiefang Guo, Qilin Zhu, Liangjing Wang, Xiaohua Shi

**Affiliations:** ^1^ Department of Gastroenterology, Suzhou Science and Technology Town Hospital, Jiangsu, China; ^2^ Department of Endoscopy, Eastern Hepatobiliary Hospital, Second Military Medical University, Shanghai, China

**Keywords:** endoscopic ultrasound, fine needle aspiration (FNA), pancreatic cystic lesion, cross sectional imaging, clinical significance, cytology, cyst fluid analysis

## Abstract

**Background and aims:**

Endoscopic ultrasound (EUS) is playing a more and more important role in the management of pancreatic cystic lesion (PCLs). The aim of our study was to evaluate the clinical impact of EUS and EUS guided fine needle aspiration (FNA) on patients with low-risk PCLs.

**Materials and methods:**

Low-risk PCL patients who underwent EUS-FNA in 2 edoscopic centers were retrospectively collected and analyzed. The clinical impact of EUS-FNA on these patients was analyzed and the predictors for significance EUS-FNA (defined by diagnosis and treatment method change, new high-risk feature identified after imaging scans) were analyzed by logistic regression analyses.

**Results:**

From July 2004 to February 2017, 186 patients with low-risk PCLs were included. The study cohort had a mean age of 52.4 ± 15.9 years (range: 19-86 years) with 89 (47.8%) male patients included. The clinical significance of EUS-FNAs was observed in 74 patients (39.8%). The presumed diagnoses of PCLs by imaging were changed in 51 (51/74, 68.9%) patients. Nineteen (19/74, 25.7%) new high-risk features were identified by EUS-FNA, and four patients (4/74, 5.4%) underwent surgery due to suspicious or malignant cytology. Based on multivariate analysis, large cyst size [odds ratio (OR): 1.12, 95% confidence interval (CI): 1.02–1.19, P = 0.033], young age (OR: 0.94, 95% CI: 0.91–0.99, P = 0.041) and BMI over 25 (OR: 3.15, 95% CI: 1.29–7.86, P = 0.013) were independent predictors of clinical significance for EUS-FNA. The optimal age and cyst size to predict significance EUS-FNA was 46.0 years and 2.3cm.

**Conclusions:**

On the basis of a 2-center retrospective study, EUS-FNA was clinically significant in about 40% of low-risk PCLs, especially in young, large cyst size, and overweight patients.

## Introduction

Pancreatic cystic lesions (PCLs) are being incidentally detected at an increased rate due to the widespread use of cross-section imaging technologies (1). The incidence of PCLs ranged from 1.9% to 13.5% in different races, and there was a strong correlation between increasing age and the prevalence of PCLs (2).

PCLs represent a wide clinicopathologic spectrum. Most PCLs are benign, but mucinous neoplasm, including mucinous cystic neoplasm(MCNs) and intraductal papillary mucinous neoplasm (IPMNs) represent precursor lesions to invasive adenocarcinoma (3). Surgical resection plays a core role in the management of PCL. However, the surgical indications are varied between different guidelines (4–6). Furthermore, following these guidelines resulted in an inaccuracy in determining the true surgery candidates (7, 8). Therefore, objective indications obtained by advanced modality is needed to better manage PCLs.

Endoscopic ultrasound (EUS) and EUS-guided fine needle aspiration (FNA) play more and more important roles in the management of PCLs. EUS enables us to show more detailed cyst features, and the objects indicators in cyst fluid such as cytology, biochemical markers, and molecular bio-makers obtained by EUS-FNA can further help us identify the nature of PCLs (9). Similar with the recommendations for surgical indications, the indications for EUS and EUS-FNA also vary between different guidelines (4–6). In general, EUS is recommended only when a PCL has high risk factors. However, only 6% of PCLs had high risk features when identified (10). Although most PCL related pancreatic cancers occur in cysts with high risk features, the pancreatic cancer related mortality in low-risk cysts should not be ignored (11, 12).

Although EUS-FNA is an invasive procedure, it has been identified to be safe (13). The value of EUS in the vast majority of low risk PCLs is rare studied. Whether the EUS-FNA has clinical impact on low-risk PCLs is not determined yet.

To address the aforementioned issues, we conducted this 2-center retrospective study to explore the value of EUS and EUS-FNA in PCL patients who had no imaging high-risk features. The more precise indications to perform EUS-FNA in low-risk PCL patients are also explored in this study.

## Materials and methods

### Patients selection

From July 2004 to February 2017, the PCL patients who performed EUS-FNA in Eastern Hepatobiliary Hospital, Shanghai, China and Suzhou Science and Technology Town Hospital, Jiangsu province, China, were retrospectively analyzed. The indications for EUS-FNA in our study were as follow: (1) EUS performed before the indications were clarified in guidelines; (2) The cross-sectional imaging diagnoses were not determined; (3) We intended to perform EUS guided ethanol ablation after malignancy was excluded by EUS-FNA; (4) The patient himself requested further scans to alleviate anxiety. All patients had EUS under the supervision of an anesthesiologist, and prophylactic antibiotics were given to all patients prior to EUS-FNA. All of the procedures were carried out by experienced therapeutic endoscopists who completed more than 100 EUS scanning cases each year. EUS was performed using linear echoendoscopes (GIF-UCT260; Olympus Corporation, Japan or SP-900,Fuji Corporation, Japan). Cyst fluid were aspirated under EUS guidance using a 19-gauge or 22-gauge needle (NA-200H-8022; Olympus Corporation or Expect; Boston Scientific, Massachusetts, MA or Echo Tip Ultra; Cook Medical, IN). The morphology of the cyst was evaluated and recorded by endoscopists. The cystic fluid was evaluated by the “string sign” initially and then sent for cytology evaluation and assessment of the carcino-embryonic antigen (CEA), glucose (year after 2016), and amylase levels. We used elevated cyst fluid CEA levels >192ng/mL, a positive string sign or a cyst fluid glucose < 25 mg/dl to define mucinous cysts. The serous cyst was diagnosed by clear non-viscous cystic fluid with negative string sign and cystic fluid CEA levels <192ng/mL in multilobulated cyst. The pseudocyst was diagnosed by elevated amylase and lipase in cyst fluid with negative string sign and cystic fluid CEA levels <5ng/mL. Cytology was considered as the golden standard for malignancy. All EUS-FNA were performed within 3 months after the cysts were identified.

The patient inclusion criterion were as follow: (1) Patients who underwent EUS-FNA and cyst fluid analyses in these 2 centers. (2) Before EUS-FNA, the cross-sectional imaging examinations were performed. (3) The detailed medical record and follow up data can be obtained. (4) Patients without high risk features recommended by Fukuoka guidelines (low-risk PCLs) (5). The exclusion criterion were as follow: (1) Patients who had one of the following clinical or radiologic high risk features (pancreatitis, size≥3 cm, enhancing nodule, main pancreatic duct dilatation, thick cyst wall, abrupt change in main pancreatic duct diameter with upstream parenchymal atrophy, lymphadenopathy, elevated serum CA 19-9, cyst growth ≥ 5 mm/2 years) (5). (2) The cyst fluid analyses were incomplete due to insufficient cyst fluid. (3) EUS performed by trainees. (4) The aforementioned PCL high risk features in EUS reports were incomplete. (5) The medical record or follow up data can not be obtained. The study was approved by the Institutional Review Board of both hospital.

### Definition of clinical significance EUS

The primary aim of our study is to explore whether EUS or EUS-FNA has clinical significance in low-risk PCLs. All the low-risk PCLs included in our study will undergo surveillance according to the current guidelines.Therefore, we defined EUS as having clinical significance according to the following criterion: (1) The imaging diagnoses were inconsistent with the diagnoses after EUS-FNA; (2) EUS-FNA identified new high-risk features in accordance with the 2017 Fukuoka guidelines (5); and (3) The management approach was changed after EUS-FNA. That means the endoscopic or surgical treatment would be adopted after EUS-FNA.

The second aim of our study was to identify the more precise indications to perform EUS-FNA. Subjects were divided into 2 groups: the clinical significance group and clinical insignificance group.The demographic information, smoking and drinking status, body mass index (BMI), pancreatic cancer family history, presence of non-specific abdominal symptoms (abdominal pain, abdominal discomfort, weight loss), presence of diabetes mellitus, cross-sectional imaging findings (including size, location, single or multiple cyst morphology) were reviewed and analyzed.

### Statistics analysis

Continuous variables with a normal distribution are represented by means and standard deviation (SD), while categorical variables are represented by numbers (%). The Student’s unpaired t-test for normal distributed continuous variables and the Mann–Whitney U test for non-normally distributed variables were used to compare demographic data and imaging findings between the clinical significance and insignificance groups. For categorical data, we utilized the χ^2^ test, and for cell counts less than 5, we used Fisher’s exact test. The independent high risk factors related with clinical significance EUS were calculated using univariate analysis and logistic regression analysis. The optimal cutoff level of parameters were assessed by receiver operator characteristic (ROC) curve. A statistically significant P-value of less than 0.05 was used. Using the SPSS 22.0 software, the data was examined (SPSS, Chicago, Illinois, USA).

## Results

### Patients and cyst imaging characteristics

**Figure 1:**
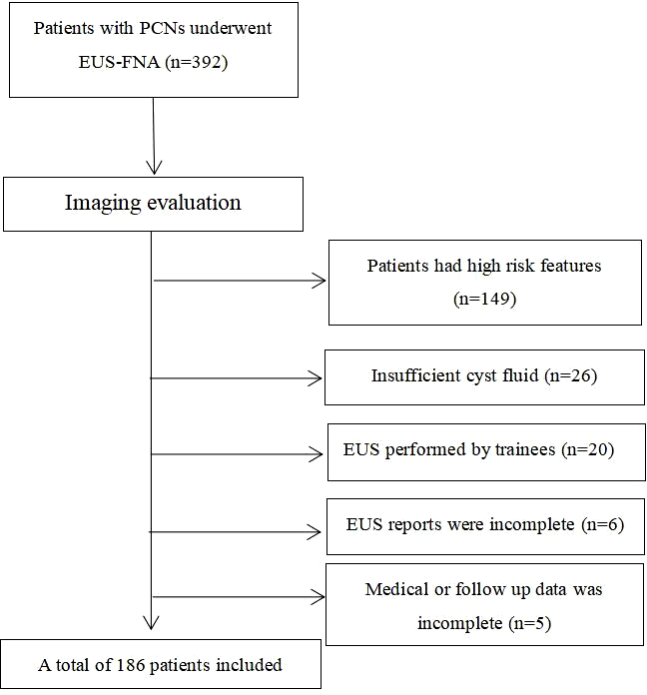
Patient selection flowchart (PCN, pancreatic cystic neoplasm; EUS-FNA, Endoscopic ultrasound guided fine needle aspiration).

From July 2004 to February 2017, a total of 392 PCL patients performed EUS-FNA in the 2 centers. After the inclusion and exclusion criterion were applied to the study cohort, a total of 186 patients with low risk cysts were included in our study (**Figure 1**). The demographic data and cyst imaging characteristics were summarized in **Table 1**. The study cohort had a mean age of 52.4 ± 15.9 years (range: 19-86 years) with 89 (47.8%) male patients included. According to the cross-sectional imaging results, the mean cyst size was 2.15 ± 1.80 cm. Most cysts had size larger than 2cm (124/186, 66.7%). Sixty-one (32.8%) cysts located in the head of pancreas, 70 cysts (37.6%) located in the body of pancreas and 55 (29.6%) cysts located in the tail of pancreas. Most of the cysts (156/186, 83.9%) were presented with single cyst morphology on cross-sectional imaging. Analyzing their personal life history, 52 (28.0%) patients had smoking history, 25 (13.4%) patients had alcohol abuse history and 16 (8.6%) patients had pancreatic cancer family history. The mean BMI for the study cohort was 23.47 ± 3.22 with 60 patients (32.3%) had BMI over 25. Forty-five (24.2%) had DM and 76 (40.9%) had non-specific abdominal symptoms at the time of diagnosis.

**Table 1:** Clinical and imaging characteristics of 186 pancreatic cystic neoplasm patients.

Total case number	N=186
Age(yr)± SD	52.4 ± 15.9
Sex(M:F)	89:97
Diameter, mean ± SD(cm)	2.15 ± 1.80
Diameter ≤1.0cm, n%	7 (3.8)
1.0<Diameter ≤1.5cm, n%	29 (15.6)
1.5<Diameter ≤2.0cm, n%	36 (19.4)
2.0<Diameter ≤2.5cm, n%	48 (25.8)
2.5<Diameter ≤3.0cm, n%	76 (40.9)
Location of cysts: head/body/tail, n(%)	61/70/55
Single/multiple cyst morphology	156/30
Smoking history, n(%)	52 (28.0)
Alcohol abuse history, n(%)	25 (13.4)
BMI ± SD	23.47 ± 3.22
BMI over 25, n(%)	60 (32.3)
Pancreatic cancer family history, n(%)	16 (8.6)
Presence of DM, n(%)	45 (24.2)
Presence of non-specific abdominal symptoms, n(%)	76 (40.9)

SD, standard deviation; DM, diabetes mellitus; BMI, Body Mass Index.

### The clinical significance of EUS-FNA on patients with low risk PCLs

The clinical significance EUS-FNAs were observed in 74 patients (39.8%). After EUS-FNA, the presumed diagnoses of PCLs by cross-sectional imaging were changed in 51(51/74, 68.9%) patients among the 74 patients. The most common diagnosis change was mucinous cysts (MCN and IPMN) turned out to be SCNs (36/51, 70.6%). Twelve SCNs(12/51, 23.5%) turned out to be mucinous cysts after EUS-FNA. Two SCNs (3.9%) and one MCN (2.0%) turned out to be pancreatic neuroendocrine tumor (pNET). Besides, 19 (19/74, 25.7%) new high risk features were identified by EUS-FNA, including 10 mural nodules (10/19, 52.6%), 5 (5/19, 26.3%) main pancreatic duct dilation and 4 (4/19, 21.1%) cyst size >3cm. Among the 19 patients, 8 patients underwent endoscopic ethanol ablation due to new high risk features were identified. Moreover, 4 patients (4/74, 5.4%) underwent surgery due to suspicious or malignant cytology. Finally, 2 IPMN with malignant, 1 IPMN with high-grade intraepithelial neoplasia and 1 pNET were identified by pathological examinations.

### Predictors of clinical significance EUS-FNA in patients with low-risk PCLs

The patients were divided into 2 groups, the clinical significance EUS-FNA group and clinical insignificance EUS-FNA group. **Table 2** compares the clinical, demographic, and cystic characteristics of these two groups. Patients with a clinical significance EUS-FNA were younger (48.5 ± 12.7 vs 56.0 ± 13.6, P=0.034). The cyst size was larger in clinical significance EUS-FNA group than in clinical insignificance EUS-FNA group (2.36 ± 1.54cm vs 1.93 ± 1.36cm, P=0.043). The patients who had BMI over 25 is much more in clinical significance EUS-FNA group (32/74, 43.2% vs 28/112, 25.0%, P=0.029). Moreover, patients with pancreatic cancer family history was not fully significance common in clinical significance EUS-FNA group (10/74, 13.5% vs 6/106, 5.7%, P=0.052). The gender, tumor location, cyst morphology, smoking history, alcohol abuse history and presence of DM and presence of non-specific abdominal symptoms did not differ significantly between these two groups.

**Table 2:** Comparisons of the clinical and imaging feature between significance and insignificance EUS-FNA.

	Significance (n = 74)	Insignificance (n = 112)	P value
Age (yr)± SD	48.5 ± 12.7	56.0 ± 13.6	0.034
Sex (M:F)	36:38	53:59	0.860
Diameter, mean ± SD (cm)	2.36 ± 1.54	1.93 ± 1.36	0.043
Location of cysts: head/body/tail,n (%)	25/28/21	36/42/34	0.953
Single/multiple cyst morphology	59/15	97/15	0.212
Smoking history,n (%)	20 (27.0)	32 (28.6)	0.818
Alcohol abuse history,n (%)	9 (12.2)	16 (14.3)	0.678
BMI ± SD	24.06 ± 3.42	22.65 ± 3.01	0.108
BMI over 25, n (%)	32 (43.2)	28 (25)	0.029
Pancreatic cancer family history,n (%)	10 (13.5)	6 (5.4)	0.052
Presence of DM, n(%)	14 (18.9)	31 (27.7)	0.172
Presence of non-specific abdominal symptoms, n (%)	30 (40.5)	46 (41.1)	0.942

SD, standard deviation; DM, diabetes mellitus; BMI, Body Mass Index.

The age, cyst size, BMI over 25 and pancreatic cancer family history were included in multivariate analysis, the gender was adjusted. Large cyst size [odds ratio (OR): 1.12, 95% confident interval (CI): 1.02–1.19, P=0.033], young age (OR: 0.94, 95% CI: 0.91–0.99, P=0.041) and BMI over 25 (OR:3.15, 95% CI: 1.29-7.86, P=0.013) were independent predictors of clinical significance EUS-FNA (**Table 3**).

**Table 3:** Multivariate predictors for clinical significance EUS-FNA in low-risk PCLs patients.

Parameters	OR	95%CI	P value
Gender	2.36	0.56-6.89	0.689
Cyst size	1.12	1.02–1.19	0.033
Age	0.94	0.91-0.99	0.041
BMI over 25	3.15	1.29-7.86	0.013
Pancreatic cancer family history	1.89	0.92-2.45	0.085

BMI, Body mass index; OR, odds ratio; CI, confident interval.

**Figure 2:**
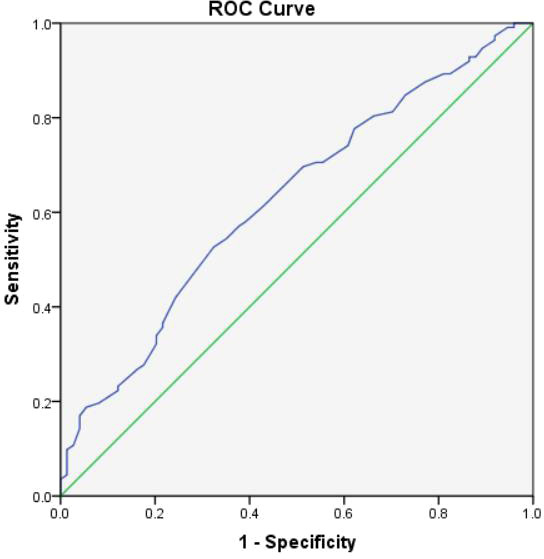
The ROC curve analysis to predict the optimal age for clinical significance EUS-FNA (the area under the curve=0.626).

**Figure 3:**
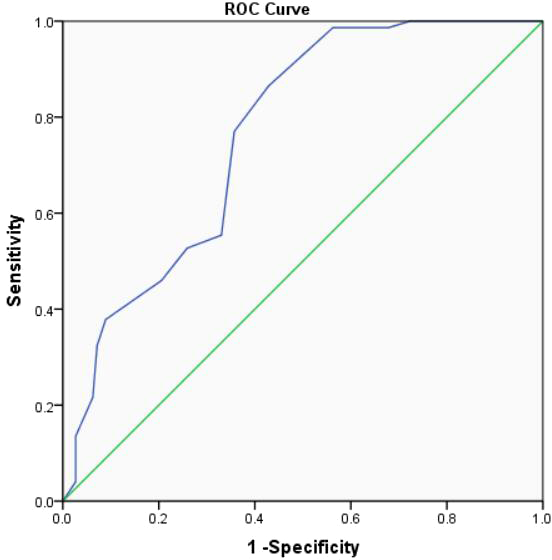
The ROC curve analysis to predict the optimal cyst size for clinical significance EUS-FNA (the area under the curve=0.765).

Moreover, according to ROC curve analyses, the optimal age to predict significance EUS-FNA was 46.0 years (**Figure 2**) and the optimal cyst size to predict significance EUS-FNA was 2.3cm (**Figure 3**).

## Discussion

PCLs are rather prevalent. However, there is still a debate about how PCLs should be managed. The risk of malignancy should be carefully assessed. However, due to morphological overlap and the lack of unique imaging characteristics, a precise diagnosis of PCNs might be challenging (14, 15).

EUS and EUS based techniques are more and more common used in the management of PCLs (9). EUS is particularly useful for assessing diagnostic features and potential predictors of malignancy in PCLs. The performance of cytology in differentiating malignant and benign PCLs and the cystic fluid CEA level in differentiating mucinous and non-mucinous PCLs are sub-optimal (16). However, due to the low prevalence of malignancy in incidental PCLs and the invasive nature of the procedure, the indications to perform EUS-FNA tend to be conserved in current guidelines. Only a small percentage of patients will be recommended to perform EUS-FNA according to the recommendations. The vast majority of PCLs without high-risk features still harbor malignant PCLs. How to find these malignant patients is a clinical challenge with little focus on it. In this study, we identified EUS-FNA maybe useful in 39.8% low risk PCL patients. Additionally, we found that a cyst size larger than 2.3 cm, age<46 at the diagnosis, and BMI over 25 at the diagnosis are predictors of clinical significance EUS-FNA. Our study provides further evidence for the important role of EUS-FNA in the management of PCNs and identifies which patients with low-risk PCNs might benefit from EUS-FNA.

The value of EUS and EUS-FNA in patients with PCLs has been studied in many previous studies. According to the 2 studies by Allen et al. and Ferrone et al., EUS alone influences the management of 40% of PCNs discovered by chance (17, 18). The role of EUS-FNA was further explored. Ardengh et al. concluded that EUS-FNA has a management impact in almost 72% of incidental PCLs through a large prospective study. They deemed EUS-FNA had a major influence on the management strategy (19). In 2016, Rodríguez-D’Jesús et al. reported that EUS-FNA altered the diagnosis and management in 39% and 21% of patients with PCLs through a retrospective study. They concluded that EUS and EUS-FNA impact the diagnosis and management of patients with PCLs; therefore, both are necessary in the workup of these patients (20). In 2019, Chang et al. Conducted a large sample size retrospective study and reported that EUS-FNA changed the diagnosis in 45.7% PCLs patients and 54.5% patients with presumed branch duct (BD) IPMN and changed the management recommendation in 35.6% of patients with PCLs and 50.5% patients with BD-IPMN (21). Additional, they concluded EUS-FNA may be more useful in younger patients and in patients with a cyst size larger than 3cm (21). However, all the aforementioned studies did not focus on patients with low-risk PCLs. Due to the higher difficulty in making accurate diagnosis in low-risk PCLs, we deemed the composition of the study cohort is one of the highlights of our study.

In the past, EUS-FNA for PCLs was reported to be associated with high incidence of complications. However, the situation changed recently. According to the recent meta-analysis (13, 22) and prospective randomized trial (23), we concluded that EUS-FNA is a safe procedure for diagnosing PCL that has a low risk of complications. The majority of side effects were minor, self-limiting, and did not necessitate medical attention. Extending the indications for EUS-FNA is logically possible and do not increase risk.

To make a definitive diagnosis for patients without adequaate pathological specimen seems impossible. The value of cytology obtained by EUS-FNA is limited in differentiating MCNs and SCNs with 42% sensitivity (24). Only about 35% of cytologic samples obtained by EUS-FNA were informative (25). Hence, the incidence of changing diagnoses after EUS-FNA is not included in our study. Instead, the clinical significance is adopted by us to assess the clinical impact of EUS-FNA on patients with low-risk PCLs. A more positive or conservative approach will be adopted after clinically significant EUS-FNA, including follow-up strategy and treatment strategy.

Many progresses have been described into the field of EUS-FNA for PCLs. For example, the intracystic Glucose level is reported to be more accurate than CEA level in diagnosing mucinous cysts (26, 27). The reported diagnostic yield for EUS-guided through the needle microforceps biopsy (EUS-TTNB) in diagnosing a specific cyst type was significantly higher with TTNB histology (72.5%) compared to that in FNA cytology (38.1%) (28). The *in vivo* imaging of the cyst epithelium can be visualized using EUS-guided needle-based confocal laser endomicroscopy (cCLE). Antonio et al. found that the diagnostic yield for nCLE at a US facility was 84.1%, much higher than the current “composite standard” (clinical, morphological, cyst fluid cytology, and chemical analyses) (29). However, the study cohort of our study is obtained between 2004 and 2017, when these techniques were not applied in clinical practise. Moreover, these techniques may not be applicable in most centers. Therefore, the conclusion of our study may be applicable to most centers.

There were certain limitations of our study. First, it was a retrospective review of a cohort of patients who underwent EUS-FNA. Follow-up data to determine which feature is associated with progression are lacking. Second, interobserver variability is unavoidable which may have caused bias. Third, the study’s sample size for each subgroup analysis was relatively small, which constrained the results. A big prospective cohort is clearly required.

## Conclusion

On the basis of a 2 centers retrospective study, EUS-FNA was clinically significance in about 40% of low-risk PCLs, especially in young, large cyst size and overweight patients. Extending the indications of EUS-FNA may be reasonable based on the conclusion of our study. However, prospective study with large sample size is needed to further verify the findings of our study.

## Data availability statement

The raw data supporting the conclusions of this article will be made available by the authors, without undue reservation.

## Ethics statement

Written informed consent was obtained from the individual(s) for the publication of any potentially identifiable images or data included in this article.

## Author contributions

SP: Data collection and statistical analysis. JL: Paper writing. JG: Design of the study and data collection. LW: Interpretation of data. QZ: Literature search. XS: Manuscript review design of the study. All authors contributed to the article and approved the submitted version.

## Conflict of interest

The handling editor [ZJ] declared a shared parent affiliation with the author [JG] at the time of review.

The remaining authors declare that the research was conducted in the absence of any commercial or financial relationships that could be construed as a potential conflict of interest.

## Publisher’s note

All claims expressed in this article are solely those of the authors and do not necessarily represent those of their affiliated organizations, or those of the publisher, the editors and the reviewers. Any product that may be evaluated in this article, or claim that may be made by its manufacturer, is not guaranteed or endorsed by the publisher.
